# Added value of body MRI to detect primary abdominal malignancies in the diagnostic work-up of patients with adenocarcinoma of unknown primary

**DOI:** 10.1007/s00330-024-11149-w

**Published:** 2024-10-29

**Authors:** Jeroen R. J. Willemse, Max J. Lahaye, Elisabeth P. Goedegebuure, Petur Snaebjornsson, Serena Marchetti, Marieke Vollebergh, Larissa W. van Golen, Wouter V. Vogel, Sajjad Rostami, Zuhir Bodalal, Regina G. H. Beets-Tan, Doenja M. J. Lambregts

**Affiliations:** 1 Department of Radiology, The Netherlands Cancer Institute, Amsterdam, The Netherlands; 2 GROW Research Institute for Oncology & Reproduction – Maastricht University, Maastricht, The Netherlands; 3 Department of Pathology, The Netherlands Cancer Institute, Amsterdam, The Netherlands; 4 Faculty of Medicine, University of Iceland, Reykjavik, Iceland; 5 Department of Medical Oncology and Clinical Pharmacology, The Netherlands Cancer Institute, Amsterdam, The Netherlands; 6 Department of Gastrointestinal Oncology, The Netherlands Cancer Institute, Amsterdam, The Netherlands; 7 Department of Nuclear Medicine, The Netherlands Cancer Institute, Amsterdam, The Netherlands; 8 Department of Radiation Oncology, The Netherlands Cancer Institute, Amsterdam, The Netherlands

**Keywords:** Neoplasms, Magnetic resonance imaging, Positron emission tomography computed tomography

## Abstract

**Purpose:**

This study aimed to evaluate the added benefit of body MRI (covering the chest, abdomen, and pelvis) to detect the primary tumour in patients with adenocarcinoma of unknown primary (ACUP) and a suspected abdominal malignancy in whom previous diagnostic work-up with CT and/or FDG-PET/CT did not yield a primary tumour diagnosis.

**Methods:**

Thirty ACUP patients with a suspected primary tumour in the abdomen/pelvis (based on pathology and/or pattern of disease) underwent MRI (T2-weighted, DWI, pre- and post-contrast T1-weighted) after completion of their initial diagnostic work-up with CT and/or PET/CT. Effects of MRI to establish a primary tumour diagnosis (and to detect additional metastatic sites) were documented. Integration of all available imaging data, additional diagnostic procedures (e.g., endoscopy), histopathology, and whole genome sequencing served as the composite standard of reference.

**Results:**

MRI rendered a possible primary tumour diagnosis in 16/30 (53%) cases, which aligned with the final clinical diagnosis in 9/16 (56%) of these cases, thus resulting in a confirmed primary tumour diagnosis in 30% of our total patient cohort. These included four gastrointestinal, two hepatobiliary, one pancreatic, one ovarian and one breast cancer. MRI revealed extra metastatic sites in five patients (17%).

**Conclusion:**

MRI can be of added value in the diagnostic work-up of ACUP patients with a suspected primary tumour originating from the abdomen or pelvis, in particular to detect gastrointestinal or hepatobiliary malignancies. Larger studies are needed to confirm these results and identify specific ACUP patients that are most likely to benefit from MRI.

**Key Points:**

***Question*** *Can body MRI help identify the primary tumour in patients with adenocarcinoma of unknown primary (ACUP)?*

***Findings*** *In this pilot of n* *=* *30 ACUP patients with clinically suspected abdominal malignancies, body MRI was able to establish the primary tumour in 30% of cases.*

***Clinical relevance*** *Body MRI can be of added value (as an adjunct to CT and/or PET/CT) in the diagnostic work-up of ACUP patients with a suspected primary tumour originating from the abdomen or pelvis, especially to detect gastrointestinal or hepatobiliary malignancies.*

## Introduction

Cancer of unknown primary (CUP) represents a heterogeneous collection of disseminated cancers in which, despite routine diagnostic procedures, the primary tumour remains unidentified. An estimated 3–5% of all newly diagnosed cancers can be classified as CUP, of which the majority (80%) are adenocarcinoma of unknown primary (ACUP) [1].

The prognosis of ACUP patients is poor, with estimated median survival rates of 4–6 months [2, 3]. Identifying the primary tumour is essential in providing targeted treatment options, thereby increasing survival, but this remains a continuous challenge. Based on autopsy studies, primary tumours are mostly located in the lung, pancreas, and hepatobiliary system [4, 5].

Guidelines from the European Society of Medical Oncology (ESMO) state that the minimal diagnostic work-up for (A)CUP patients should include physical examination, basic blood and biochemical analyses, and contrast-enhanced CT, or MRI, of the neck, thorax, abdomen, and pelvis. The clinical presentation, pathology results, and the pattern of known metastatic sites guide the choice of additional diagnostic examinations, such as endoscopic procedures or tumour-specific laboratory markers [6]. Whole-body 18[F]2-fluoro-2-deoxy-D-glucose (FDG) positron emission tomography with low-dose CT (FDG-PET/CT) is an optional but commonly used second-line imaging tool in ACUP. It has benefit in finding additional metastases and depicting the true extent of disease. Moreover, FDG-PET/CT has shown value in detecting primary head and neck (H&N) cancers in patients presenting primarily with squamous cell cervical lymph node metastases [6]. MRI is mainly performed using dedicated protocols for H&N, brain metastases, or pelvic malignancies.

Owing to the expanding number of diagnostic tools at the physicians’ disposal, primary tumour detection rates have improved [7]. Nevertheless, the primary tumour remains occult in a significant proportion of patients [4]. Overall, FDG-PET/CT has been reported to detect an underlying primary tumour in ± 35–43% of CUP cases [8–10]. Evidence on the potential value of body MRI in the setting of CUP is scarce, with only a few exploratory reports [11, 12]. Previous studies have shown benefit for whole-body MRI (with full body coverage) in different oncological settings, including cancer screening in high-risk patients or for staging specific cancer types such as multiple myeloma and prostate cancer [13–15]. The main benefit of MRI is that it combines high-resolution anatomical imaging with functional imaging sequences such as diffusion-weighted imaging (DWI) which has shown great value in increasing the sensitivity of MRI to detect malignant disease [16, 17].

In our institution, a tertiary referral centre for ACUP patients, body MRI covering the chest, abdomen, and pelvis is offered as an optional additional tool after CT and PET/CT in the diagnostic work-up of ACUP since January 2022. It is selectively applied in patients in whom neither CT nor PET/CT was able to clearly establish a primary tumour diagnosis and when—based on pathology outcomes and the pattern of known metastatic disease sites—the primary tumour is suspected to be located in the abdomen or pelvis. This pilot study aimed to evaluate the potential added benefit of body MRI in this specific clinical setting, i.e., to detect the underlying primary tumour in ACUP patients after negative or inconclusive (PET/) CT findings.

## Methods

Since January 2022, our institution has routinely offered the option to perform body MRI as an adjunct clinical tool in the diagnostic work-up of ACUP patients. Data on the outcomes of these MRIs were prospectively collected till December 2023 (a 2-year pilot period) and retrospectively analysed. This study was approved by the local institutional review board, and informed consent was waived.

### Patient selection

From our institutional database, we identified all patients with a clinical diagnosis of ACUP who underwent body MRI as part of their clinical diagnostic work-up between January 2022 and December 2023. Further inclusion criteria were as follows: (1) histopathologically (biopsy) confirmed adenocarcinoma, (2) availability of previous cross-sectional imaging examinations (contrast-enhanced CT and/or FDG-PET/CT) showing the presence of metastases, and (3) no confirmed diagnosis of an underlying primary tumour based on previous CT or PET/CT. Patients who were already receiving (systemic) treatment at the time of referral or were in too poor clinical condition to undergo additional diagnostic tests were excluded. The choice to perform MRI was discussed by a dedicated multidisciplinary team (MDT) including oncologists, pathologists, nuclear medicine physicians, and radiologists. The decision was guided by all available clinical information, including histopathology, tumour markers and previous imaging findings. Patients with a suspected primary tumour in the abdomen or pelvis were selected to undergo body MRI.

### MRI protocol

All body MRI examinations were performed at 3 T (Achieva or Ingenia, Philips Healthcare). The basic protocol consisted of axial and coronal T2-weighted sequences, an axial b0 and b1000 diffusion-weighted sequence (including apparent diffusion coefficient maps), and non-enhanced and gadolinium-enhanced fat-suppressed T1-weighted sequences covering the chest, abdomen, and pelvis. At the discretion of the radiologist (and based on the available clinical information), the basic protocol could be supplemented with additional sequences of the pelvis (high-resolution sagittal, coronal, and/or axial T2-weighted series in suspected gynaecological or anorectal tumours) or upper abdomen (multiphase contrast-enhanced sequences in suspected hepatobiliary or pancreatic malignancies). As a preparatory step, patients were asked to drink 1 L of pineapple juice an hour before the scan to suppress the signal of bowel content on DWI, as described in previous protocols [18]. Patients received Buscopan (20 mg) for intestinal tonus and motility reduction.

### Previously conducted imaging examinations (CT and PET/CT)

Considering that our institution is a tertiary referral centre for ACUP patients, previously performed CT scans and FDG-PET/CT scans were mainly acquired at the referring hospitals and performed in line with national guidelines and local institutional protocols. In general, CT scans were acquired with a slice thickness of 1 mm and routinely included a portal venous phase (± 70 s post-IV injection of iodine-based contrast). Reconstructed slice thickness depended on local protocols and varied between 3 and 5 mm. FDG-PET/CT scans routinely covered the skull base to upper thighs and included a low-dose, non-enhanced CT scan for anatomical correlation and attenuation correction.

### Image evaluation

All CT, FDG-PET/CT, and body MRI examinations were prospectively evaluated and reported by board-certified radiologists or nuclear medicine physicians as part of the routine clinical diagnostic work-up using free text reports. Images were reported in a non-blinded fashion, taking into account all clinical information that was available at the time of image reporting, including pathology results, tumour markers, findings of previous imaging and/or clinical examinations, etc. Images retrieved from referring centres were generally re-evaluated and reported by a radiologist or nuclear medicine physician from the referral (and principal investigating) centre.

Two independent observers (J.W., E.G.) analysed the imaging reports and converted these into a standardised score using a scoring form designed for this study. This scoring form divided the body into the following regions: cervical lymph nodes, thoracic lymph nodes, upper abdominal lymph nodes, lower abdominal lymph nodes, inguinal lymph nodes, brain, breast, lung/pleura (incl. pleural effusion), liver, bile duct/gallbladder, pancreas, spleen, adrenal gland, kidney, urinary tract, small bowel, colon, anorectum, peritoneum (incl. ascites), prostate, uterus/ovaries, bone, chest and abdominal wall, soft-tissue, and miscellaneous. For each region, it was reported whether the diagnostic reports indicated the (possible) presence of tumour lesion(s) within this region. Finally, it was documented whether the report included a diagnosis of a possible underlying primary tumour. The confidence level with which a primary tumour diagnosis (if any) was established was graded using a three-point Likert scale: 1 = possible (low confidence), 2 = probable (moderate confidence), and 3 = highly likely (high confidence). If patients previously underwent both FDG-PET/CT and contrast-enhanced CT, the findings of both examinations were combined into a single score to represent the findings of all clinical imaging procedures performed prior to the MRI. In case of uncertainties or discrepancies between the findings documented by the two independent observers, a third independent reader (D.L. or M.L.) was consulted to reach consensus.

### Final clinical diagnosis (standard of reference)

The final primary tumour diagnosis was achieved through the integration of all imaging, additional diagnostic procedures (e.g., endoscopy), and other lab and clinicopathological data. These data were assessed by the MDT including an oncologist (S.M. or M.V.), nuclear medicine physician (L.v.G. or W.V.), radiologist (D.L. or M.L.) and pathologist (P.S.). The reference standard included biopsy results with histopathology and whole genome sequencing (WGS), if available. The level of certainty was classified in consensus by the MDT as ‘highly likely’ (> 75%) if results of pathology/WGS were in line with a clear diagnosis of a matching primary tumour on imaging and/or other diagnostic modalities, ‘probable’ (50–75%) if either imaging or histopathology/WGS provided a clear diagnosis but the other was ambiguous, and ‘possible’ if a primary tumour diagnosis was suspected on imaging OR pathology/WGS but could not be confirmed by the other modality. Patients were recorded as confirmed CUP cases if no final diagnosis could be established.

### Data analysis

The findings of body MRI were documented and compared to those of contrast-enhanced CT and FDG-PET/CT (further referred to together as (PET/) CT) on a per-region basis using descriptive analyses. The primary tumour diagnosis as established with MRI (if any) was compared to the final standard of reference.

## Results

### Patients

From January 2022 until December 2023, 106 CUP patients were referred to our institution and analysed by our local MDT. Thirteen of 106 patients were excluded from the current analysis because of a histopathological subtype other than adenocarcinoma (*n* = 6), strong suspicion of a primary tumour diagnosis at the time of referral (*n* = 6), or because additional imaging was not feasible due to poor clinical condition (*n* = 1).

After reviewing all clinical, histopathological, molecular, endoscopic, laboratory, and imaging information, the multidisciplinary team advised additional MRI in 30 of the remaining 93 patients (32%).

Our cohort of 30 patients included 18 females and 12 males with a mean age of 65 (± 11.2) years. Key patient characteristics at the time of referral are summarised in Table 1. One patient underwent only FDG-PET/CT (and no contrast-enhanced CT) prior to MRI; the remaining 29 patients underwent both contrast-enhanced CT and FDG-PET/CT. In the majority of patients (87%), imaging from referring centres was re-evaluated and reported by radiologists and nuclear medicine physicians from our institution; in the remaining 13% of cases, the original imaging reports from the referring centres were analysed. The median time interval between MRI and the most recent previous (PET/)CT was 36.5 days (range: 13–77 days).

**Table 1:** Baseline patient characteristics

		*N*	%
TOTAL		30	100%
Gender			
Female		18	60%
Male		12	40%
Age (mean ± SD)		65 (± 11.2) years	
Type of adenocarcinoma			
Mucinous		5	17%
Non-mucinous/unspecified		25	83%
Known tumour sites		Total *n* = 106	100%
Lymph nodes			
Cervical		9	9%
Thoracic		12	11%
Upper abdominal		18	17%
Lower abdominal		5	5%
Inguinal		1	1%
Lung/pleura (incl. effusion)		18	11%
Peritoneum (incl. ascites)		10	8%
Liver		8	8%
Bone		5	5%
Small bowel		3	3%
Adrenal gland		3	3%
Uterus/ovary		3	3%
Brain		2	2%
Kidney		2	2%
Thyroid		2	2%
Muscle		1	1%
Anorectum		1	1%
Bladder/urinary tract		1	1%
Penis		1	1%
Thoracic wall		1	1%

The basic body MRI protocol was used in 17 patients; 13 patients underwent the basic protocol with additional sequences, as outlined in Table 2.

**Table 2:** MRI protocol

Basic protocol (performed in all patients)					
	Single shot EPI-DWI	T2W TSE	T2W TSE	T1W 3D FFE (non- & contrast-enhanced^a^)	T1 3D FFE (contrast-enhanced^a^)
Slice orientation	Transverse	Transverse	Coronal	Transverse	Coronal
TR (ms)	Shortest	1500	1500	Shortest	Shortest
TE (ms)	Shortest	87	87	Shortest	Shortest
Acquired in plane voxel size (mm)	4.5 × 4.5	1 × 1	1 × 1	1.5 × 1.5	1.5 × 1.5
Reconstructed in plane voxel size (mm)	2.2 × 2.2	0.9 × 0.9	0.6 × 0.6	1.0 × 1.0	0.7 × 0.7
Number of stacks	3	2	3	3	2
Number of slices per stack	50	68	46	80	90
Acquired slice thickness (mm)	5	4	5	5	5
Reconstructed slice thickness (mm)	5	4	5	2.5	2.5
Slice gap	0	0.4	0.5	−2.5	−2.5
Signal averages	1	1	1	1	1
Parallel imaging factor	2.5	4	4	2	2
Fat suppression	STIR	N/A	N/A	Dixon	Dixon
b-values	0, 1000	N/A	N/A	N/A	N/A
Turbo/TSE factor	N/A	76	84	N/A	N/A
EPI factor	29	N/A	N/A	N/A	N/A
Acquisition time (per stack)	3:22	1:42	1:09	00:12	00:22
Total acquisition time	10:06	3:24	3:27	00:36	00:44

Additional protocols/sequences (performed in 13 patients^b^)					
Protocol/sequence					No. of patients
**Dedicated upper abdominal sequences (pancreas, liver):** - Multiphase post-contrast T1W Dixon^c^ (non-enhanced, arterial, portal venous, late phase) - DWI (b-value 0 & 800 s/mm^2^)					10^b^
**Dedicated multiplane high-resolution 2D pelvic imaging sequences (rectum, gyn, prostate):** - T1W; T2W (2.5–3 mm slice thickness) - DWI (b-value 0 & 800–1000 s/mm^2^)					2
**Dedicated multiplane high-resolution 2D neck/cervical spine sequences:** - T1W TSE; T2W TSE (SAG, TRA; slice thickness 1–3 mm) - T2W STIR (slice thickness 3–4 mm) - DWI (b-value 0 & 1000 s/mm^2^)					2^b^

*TR* repetition time, *TE* echo time, *EPI* echo planar imaging, *TSE* turbo spin echo, *FFE* fast field echo, *STIR* short tau inversion recovery

^a^ Acquired 2 min after intravenous administration of 15 mL gadoterate meglumine (0.5 mmol/mL Dotarem®; Guerbet)

^b^ One patient underwent additional sequences of both the upper abdomen and neck

^c^ Images acquired in multiple stacks were merged for viewing purposed

### Final diagnosis

In seventeen out of thirty patients (57%), a final diagnosis was established (13 highly likely, 2 probable, 2 possible); these included six gastrointestinal, three hepatobiliary, three pancreas, three ovarian, one lung and one breast cancer. In the remaining 13 patients (43%), integration of all available diagnostic results did not result in a final primary tumour diagnosis, leading to a confirmed diagnosis of CUP. Supplement 1 provides details of the diagnostic work-up leading to the final diagnosis of all study patients, including the findings of CT, FDG-PET/CT, body MRI, and pathology (including WGS) outcomes of all study patients.

### Findings of body MRI

**Fig. 1:**
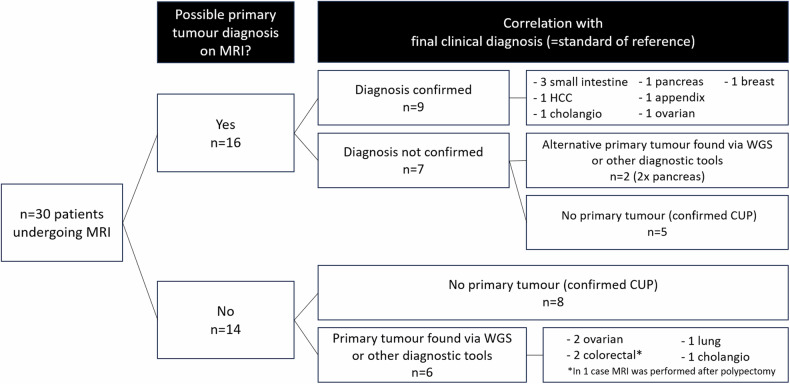
Flowchart showing the ability of MRI to detect an underlying primary tumour and its correlation with the final clinical diagnosis as established after integration of all available imaging, additional diagnostic procedures (e.g., endoscopy), and other lab and clinicopathological data

Table 3 provides an overview of the findings of MRI compared to previous imaging. Figure 1 provides a schematic overview of the performance of MRI to detect the primary tumour.

**Table 3:** MRI findings compared to those of previous (PET/)CT

Primary tumour diagnosis		
	Patients (total *n* = 30)	Location/lesion type
No change—neither (PET/)CT nor MRI diagnosed a (possible) primary tumour	14 (47%)	N.A.
MRI newly diagnosed a (possible) primary tumour not identified on (PET/)CT	8^a^ (27%)	- Gallbladder (*n* = 2) - Duodenum (interpreted on PET/CT as possible renal lesion) - Intrahepatic bile ducts - Pancreas (interpreted on PET/CT as lymph node) - Ovary - Appendix - Breast
MRI diagnosed a (possible) primary tumour that was already detected as a lesion but not interpreted to be the primary tumour on (PET/)CT	4^b^ (13%)	- Duodenum (*n* = 2) - Liver - Intrahepatic bile ducts
MRI confirmed a previous equivocal (PET/)CT diagnosis of a (possible) primary tumour	4^c^ (13%)	- Duodenum (*n* = 2) - Lung - Kidney

Additional effects of MRI		
	Patients (total *n* = 30)	Location/lesion type
MRI detected additional metastases	5 (17%)	- Peritoneum (*n* = 3) - Bone - Kidney
MRI converted (possible) tumour lesions detected on (PET/)CT to a benign diagnosis	6 (20%)	- Ovary - Adrenal gland - Anorectum - Upper abdominal lymph node - Urinary tract - Penis

^a^ Five out of eight in line with final diagnosis; for further details, see Supplement 1

^b^ Two out of four in line with final diagnosis; for further details, see Supplement 1

^c^ Two out of four In line with final diagnosis; for further details, see Supplement 1

#### Primary tumour diagnosis

In total, MRI suggested a possible primary tumour in 16/30 cases. In nine out of these sixteen cases, this diagnosis was in line with the final clinical diagnosis resulting in a confirmed MRI diagnosis in 9/30 (30%) of our total patient cohort. These nine confirmed MRI diagnoses consisted of three small intestine carcinomas, one hepatocellular carcinoma, one cholangiocarcinoma, one pancreatic cancer, one appendiceal cancer, one breast and one ovarian cancer. In the remaining 7/16 cases, the suggested MRI diagnosis could not be confirmed; in two of these cases, an alternative diagnosis was found (both pancreatic cancers), and five were confirmed CUP cases. When looking at the subgroup of seventeen patients with a confirmed clinical primary tumour diagnosis, MRI was able to correctly detect these in 9/17 (53%).

**Fig. 2:**
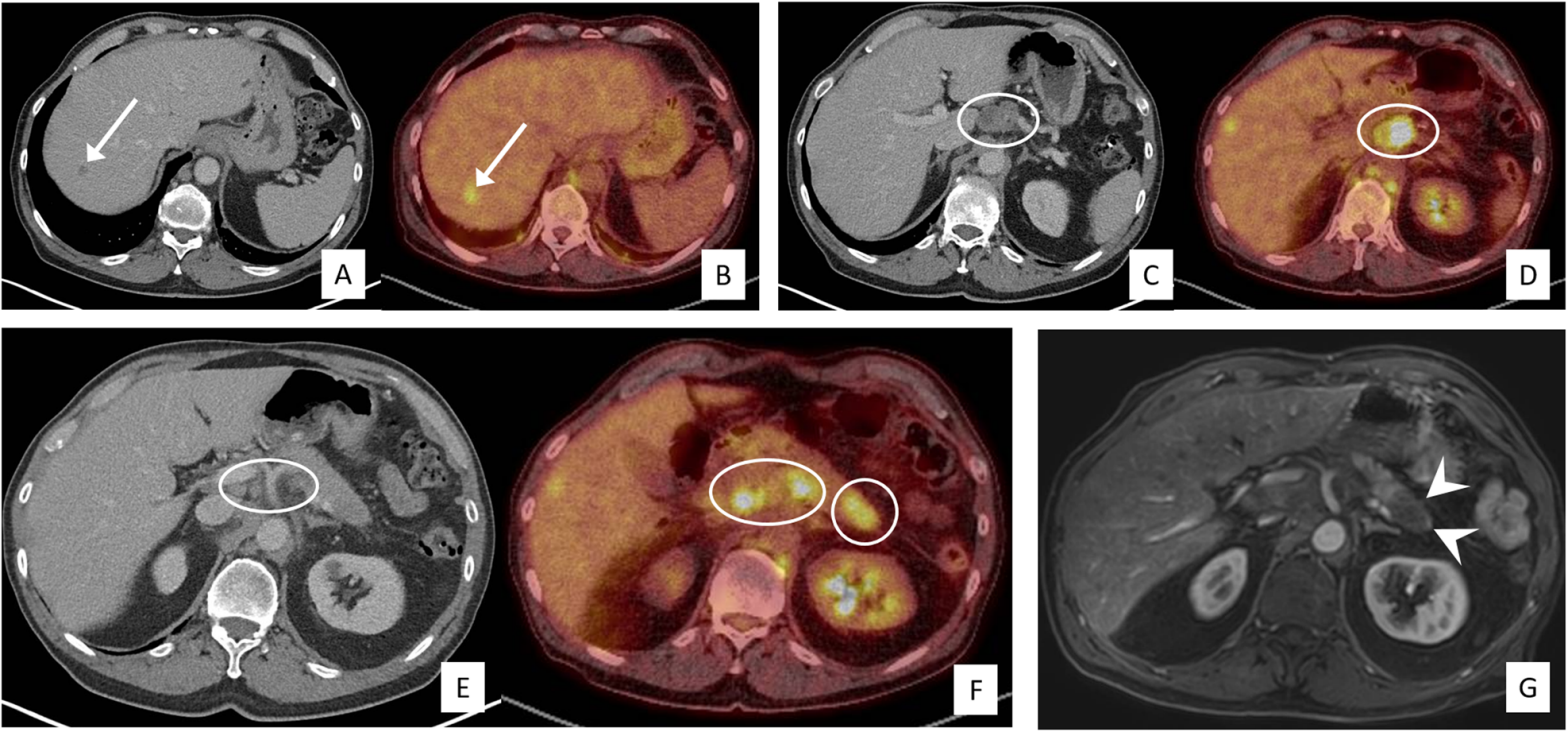
Patient (#17, see Supplement 1) diagnosed on contrast-enhanced CT and FDG-PET/CT with malignant liver lesions (arrows in **A**, **B**) and multiple pathologic upper abdominal lymph nodes (circles in **C**–**F**). On MRI, one of the lesions diagnosed on FDG-PET/CT as a peripancreatic lymph node was recognised to, in fact, concern a tumour in the pancreatic tail (arrowheads in **G**; arterial contrast-enhanced T1-weighted sequence shown). This tumour was not recognised as such on CT (**C**). This led to the correct final MRI diagnosis of a pancreatic adenocarcinoma as the underlying primary tumour

**Fig. 3:**
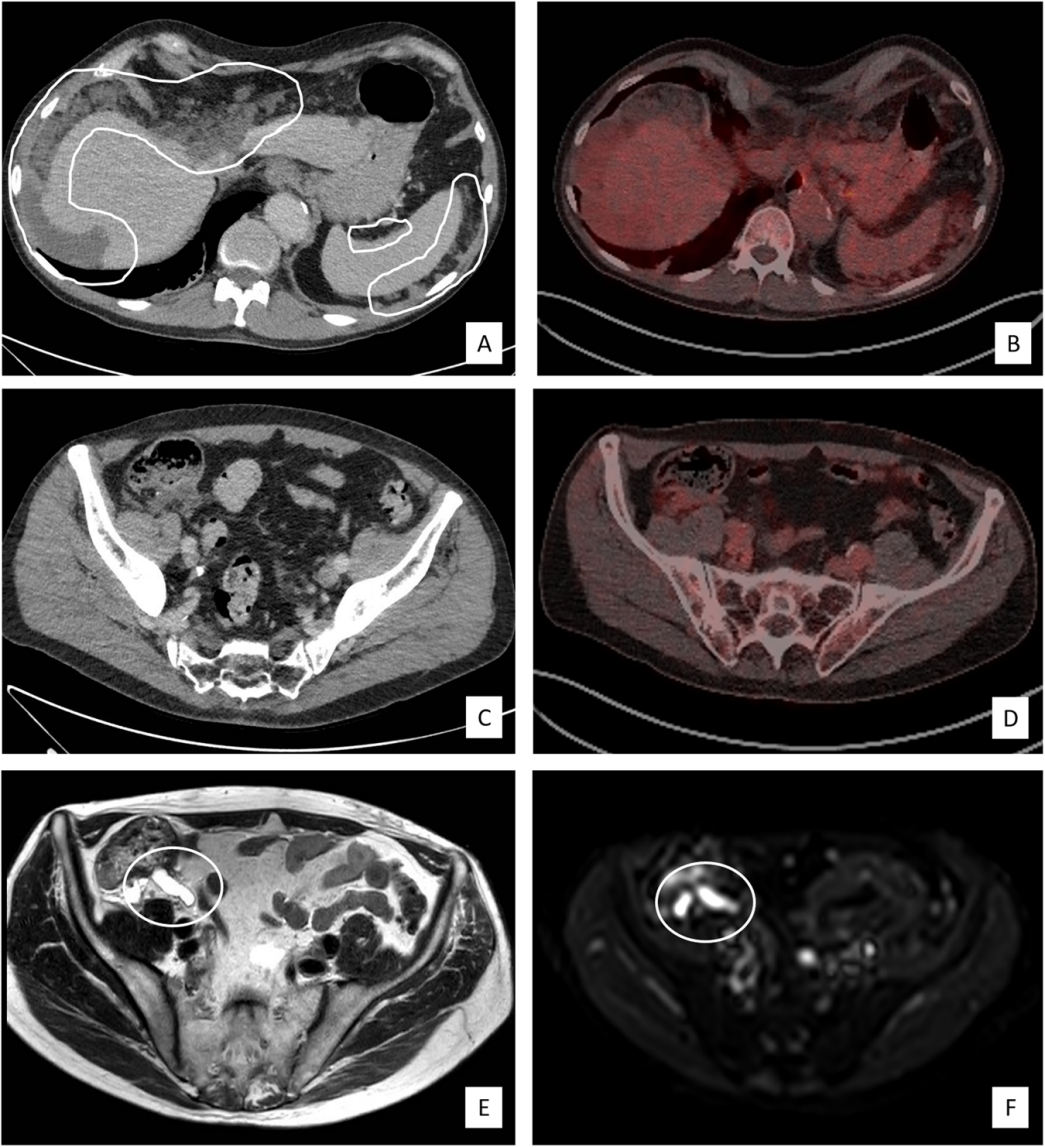
Patient (#26, see Supplement 1) diagnosed on CT with extensive peritoneal metastases in the upper abdomen around the liver and spleen (**A**); the peritoneal lesions showed only mild FDG uptake on corresponding PET/CT (**B**). No primary tumour was reported on CT (**C**) or FDG-PET/CT (**D**); no abnormalities were reported in the region of the appendix (though, in retrospect, the appendix may have been visible on CT). On T2-weighted MRI, a pathologically thickened appendix filled with fluid (mucine) was identified, which was also clearly visible on the low b-value (b0) diffusion-weighted images (circles in **E**, **F**), which led to the correct final diagnosis of a mucinous adenocarcinoma of the appendix as the underlying primary tumour

Out of the 16 (possible) primary tumours detected on MRI, four were already detected as lesions but not interpreted on (PET/)CT to represent the primary tumour (see example in Fig. 2), eight were not identified at all on previous (PET/)CT (see example in Fig. 3), and four were already suggested to be the possible primary tumour on PET/CT but with a low confidence/equivocal score. In two out of the four latter cases, MRI led to an increase in diagnostic confidence.

WGS results were already available at the time of MRI reporting in 10/30 cases.

#### Number of metastatic sites

In addition to diagnosing a possible primary tumour, MRI detected extra metastatic sites in five out of thirty patients (17%). In six patients (20%), lesions diagnosed as tumour lesions on (PET/)CT were reclassified as benign lesions on MRI. Five lesions detected on (PET/)CT (two thyroid, two brain and one intramuscular) were outside the MRI field of view; none of these concerned the primary tumour.

## Discussion

This study investigated the potential added benefit of body MRI in the diagnostic work-up of patients with adenocarcinoma of unknown primary in whom previous CT and/or FDG-PET/CT did not yet lead to a confirmed primary tumour diagnosis. MRI was selectively employed in patients in whom available clinical information (i.e., pattern of metastatic spread, tumour markers, and pathology results) raised the suspicion of a primary tumour originating from the abdomen or pelvis. In this selected subgroup, we found that adding MRI contributed to establishing a final primary tumour diagnosis in 30% of our total patient cohort.

When zooming in on the subgroup of patients with a confirmed clinical primary tumour diagnosis, MRI established a correct diagnosis in 53% of these cases. However, we should note that MRI was selectively employed in patients in whom—based on available clinical information—the primary tumour was already expected to originate from the abdomen or pelvis. Moreover, in one-third of the cases, WGS results were already available at the time of MRI reporting. These factors allowed for a better informed and more focused imaging assessment. WGS currently has an undefined role in the diagnostic work-up of CUP patients, but preliminary evidence suggests that when integrated into the diagnostic work-up, it could classify 68% of primary cancers [19]. Although genetic profiles based on WGS may strongly point towards a primary tumour, ambiguous profiles regularly emerge (as was also the case in various patients in our cohort, demonstrated in Supplement 1), stressing the need for further diagnostic confirmation of potential primary tumours suggested by WGS.

It is well known that MRI, in particular MRI with DWI, is an effective tool for detecting various types of abdominopelvic malignancies [13]. The MRIs in our study were prospectively reported (as part of clinical routine) without explicit mentioning of the respective contribution of different imaging sequences. As such, it was unfortunately not feasible to investigate the respective benefit of DWI compared to the other imaging sequences in our protocol, though we are confident that the addition of DWI made a significant contribution. In a previous small study by Gu et al investigating the screening potential of MRI with DWI in patients with CUP, MRI was able to detect a primary tumour in 23/34 (68%) patients. This cohort, however, was skewed toward patients with bone metastases that predominantly originated from primary lung cancer [20]. Another study compared FDG-PET/MRI to FDG-PET/CT in a small cohort of 20 CUP patients and found comparable diagnostic ability for the detection of primary tumours and metastases, with superior results for PET/MRI in the detection of H&N primary tumours and for PET/CT in the detection of primary pulmonary tumours [11]. Several of the tumour lesions detected with MRI but missed on previous (PET/)CT in our study were peritoneal metastases, for which MRI with DWI has a known good sensitivity [21, 22]. Six out of nine (67%) confirmed primary tumours detected with MRI were located in the gastrointestinal (GI) or hepatobiliary tract. Especially GI tumours may be difficult to discern on FDG-PET/CT as they may be obscured by physiological FDG uptake in the bowel or misclassified due to bowel movement and/or anatomical mismatch between PET and CT [23]. MRI could also be of added value in mucinous-type adenocarcinomas, which are generally less FDG-avid [24, 25]. Although mucinous tumours may also be challenging to detect on DWI (as mucin content typically does not result in restricted diffusion), they are typically well appreciated due to their high signal on T2-weighted MRI and low b-value DWI (as shown in the example of mucinous adenocarcinoma of the appendix in Fig. 3).

In addition to MRI, PET/CT with novel radiopharmaceuticals may be employed in the diagnostic work-up of ACUP. For example, Fibroblast Activation Protein Inhibitor (FAPI) has shown promising results in different GI cancers, and is currently being studied in the setting of CUP [26, 27]. In H&N CUP, FAPI-PET/CT has already shown promising results by detecting 51% of primary tumours [28].

This study has some limitations, apart from the small sample size. First, establishing a reference standard in (A)CUP patients remains challenging, especially in patients with negative diagnostic investigations. Due to the poor prognosis of ACUP, clinical follow-up is often limited, and autopsies are rarely performed to establish a final diagnosis. Second, by merging the findings from all available prior imaging (i.e., contrast-enhanced CT and FDG-PET/CT), we could not perform independent comparisons between MRI and either prior imaging modality. Moreover, we were not able to analyse the respective benefits of DWI and the other sequences in our standard protocol, nor the diagnostic yield of the additional sequences that were performed in some patients. Most, but not all (PET/)CTs acquired in referring centres were reassessed by experts from our own centre. Finally, the non-blinded prospective clinical study setup, where images were interpreted with all available clinical information available, did not allow for an unbiased, blinded evaluation of the diagnostic performance of MRI or the other diagnostic modalities.

In conclusion, the results of this pilot study support the use of body MRI as a useful diagnostic tool in ACUP patients with a suspected primary tumour in the abdomen or pelvis, in particular to help diagnose patients with hepatobiliary or GI primary malignancies. Future research should further investigate which patients are most likely to benefit from MRI, determine the optimal imaging protocol, and assess whether MRI can compete with FDG-PET/CT as a second-line imaging modality in specific subgroups, aiming to further tailor the diagnostic approach. In addition, it would be interesting to see if MRI with full body coverage could have a potential role as a second or even first-line staging tool in the general setting of ACUP.

## Supplementary information

ELECTRONIC SUPPLEMENTARY MATERIAL

## Abbreviations

ACUP: Adenocarcinoma of unknown primary
CUP: Cancer of unknown primary
DWI: Diffusion weighted imaging
FDG: 18[F]2-fluoro-2-deoxy-D-glucose
GI: Gastrointestinal
H&N: Head and neck
MDT: Multidisciplinary team
MRI: Magnetic resonance imaging
PET/CT: Positron emission tomography/computed tomography
WGS: Whole genome sequencing
