# Supplementary material for: Added value of body MRI to detect primary abdominal malignancies in the diagnostic work-up of patients with adenocarcinoma of unknown primary
Source: Eur Radiol. 2024 Oct 29;35(5):2702–11. doi: 10.1007/s00330-024-11149-w (PMC12021699; doi:10.1007/s00330-024-11149-w)
Supplement: Supplementary file 1 — ELECTRONIC SUPPLEMENTARY MATERIAL [file 330_2024_11149_MOESM1_ESM.pdf]

Added value of body MRI to detect primary abdominal malignancies in the diagnostic work-up of patients with adenocarcinoma of unknown primary

ELECTRONIC SUPPLEMENTARY MATERIAL

Eur Radiol (2024) Willemse JRJ, Lahaye MJ, Goedegebuure EP, et al.

<table>
<tr><th rowspan="2">Case N0
Age/gender</th><th colspan="3">Imaging</th><th rowspan="2">WGS
(or histopathology)</th><th rowspan="2">Final clinical diagnosis</th><th rowspan="2">Impact of body MRI</th></tr>
<tr><th>Tumour locations on PET/CT and CT</th><th>Correlation with body MRI</th><th>DDx for primary tumour</th></tr>
<tr><td>1
49,F</td><td>Thoracic LN
Upper abdominal LN
Lower abdominal LN
Liver
Pleura
Uterus
Ovary
Peritoneum</td><td><u>Known locations:</u>
Fully concordant

<u>Extra locations on MRI:</u>
None</td><td>(PET/)CT:
Ovary or Uterus

MRI:
Liver (interpreted as intrahepatic cholangiocarcinoma)</td><td>WGS:
93% Pancreas
5% Bile ducts/gallbladder

WGS results available:
☒ Before MRI
☐ After MRI</td><td>Diagnosis:
Intrahepatic cholangiocarcinoma (IHCC)

Level of certainty:
☐ Possible (<50%)
☒ Probable (50-75%)
☐ Highly likely (>75%)

Rationale:
Intrahepatic mass on MRI with possible diagnosis (though low probability) of bile duct origin on WGS. No evidence of pancreatic lesion on imaging.</td><td>☐ None
☐ Additional tumour sites detected
☐ Confirmed (PET/)CT diagnosis of primary tumour
☒ New diagnosis of primary tumour
☒ In line with final diagnosis
☐ Not in line with final diagnosis</td></tr>
<tr><td>2
61,F</td><td>Lung
Thoracic LN</td><td><u>Known locations:</u>
Fully concordant

<u>Extra locations on MRI:</u>
Peritoneum</td><td>(PET/)CT:
None

MRI:
None</td><td>WGS:
23% Breast
20% Endometrium
11% Ovary/Fallopian tube

WGS results available:
☐ Before MRI
☒ After MRI</td><td>Diagnosis:
(Extra)ovarian cancer

Level of certainty:
☐ Possible (<50%)
☐ Probable (50-75%)
☒ Highly likely (>75%)

Rationale:
Histopathology showed low grade serous carcinoma. No clear ovarian lesion on gynaecological US. Considering the metastatic pattern (with peritoneal disease) and the histopathological findings,</td><td>☐ None
☒ Additional tumour sites detected
☐ Confirmed (PET/)CT diagnosis of primary tumour
☐ New diagnosis of primary tumour</td></tr>
</table>

				the case was interpreted as low grade serous (extra)ovarian cancer.	
3<br>72,F	Peritoneum	Known locations:<br>Fully concordant<br><br>Extra locations on MRI:<br>None (no other organs involved, but multiple additional peritoneal lesions)	**(PET/)CT:**<br>None<br><br>**MRI:**<br>None	**WGS:**<br>Not performed<br><br>**Histopathology:**<br>Adenocarcinoma (small bowel or pancreaticobilliary origin)	**Diagnosis:**<br>Confirmed CUP<br><br>**Level of certainty:**<br>N/A (no diagnosis)<br><br>**Rationale:**<br>No tumour identified on imaging that would fit the histopathologic profile.	☒ None<br>☐ Additional tumour sites detected<br>☐ Confirmed (PET/)CT diagnosis of primary tumour<br>☐ New diagnosis of primary tumour
4<br>69,M	Ascites<br>Bone<br>Pleura<br>Lung	Known locations:<br>Fully concordant<br><br>Extra locations on MRI:<br>Peritoneum	**(PET/)CT:**<br>None<br><br>**MRI:**<br>None	**WGS:**<br>Not performed<br><br>**Histopathology:**<br>Adenocarcinoma (upper GI Tract or pancreaticobilliary origin)	**Diagnosis:**<br>Confirmed CUP<br><br>**Level of certainty:**<br>N/A (no diagnosis)<br><br>**Rationale:**<br>No tumour identified on imaging that would fit the histopathologic profile.	☐ None<br>☒ Additional tumour sites detected<br>☐ Confirmed (PET/)CT diagnosis of primary tumour<br>☐ New diagnosis of primary tumour
5<br>63,F	Bone	Known locations:<br>Fully concordant<br><br>Extra locations on MRI:<br>None	**(PET/)CT:**<br>None<br><br>**MRI:**<br>None	**WGS:**<br>Not performed<br><br>**Histopathology:**<br>Adenocarcinoma (upper GI tract or pancreaticobilliary origin)	**Diagnosis:**<br>Confirmed CUP<br><br>**Level of certainty:**<br>N/A (no diagnosis)<br><br>**Rationale:**<br>No tumour identified on imaging that would fit the histopathologic profile.	☒ None<br>☐ Additional tumour sites detected<br>☐ Confirmed (PET/)CT diagnosis of primary tumour<br>☐ New diagnosis of primary tumour

6<br>63,F	Cervical LN<br>Thoracic LN<br>Upper abdominal LN<br>Thyroid<br>Lung<br>Adrenal gland	<u>Known locations:</u><br>Concordant (except for adrenal gland: benign on MRI. Thyroid not within MRI FOV)<br><br><u>Extra locations on MRI:</u><br>None	**(PET/)CT:**<br>Thyroid or lung<br><br>**MRI:**<br>Lung	**WGS:**<br>99% GI tract (small/large bowel or appendix)<br><br>WGS results available:<br>☐ Before MRI<br>☒ After MRI	**Diagnosis:**<br>Confirmed CUP<br><br>**Level of certainty:**<br>N/A (no diagnosis)<br><br>**Rationale:**<br>No tumour identified on imaging that would fit the WGS profile, negative colonoscopy.	☐ None<br>☐ Additional tumour sites detected<br>☒ Confirmed (PET/)CT diagnosis of primary tumour<br>☐ In line with final diagnosis<br>☒ Not in line with final diagnosis<br>☐ New diagnosis of primary tumour
7<br>63,F	Thoracic LN<br>Lung<br>Bone<br>Pleura	<u>Known locations:</u><br>Fully concordant<br><br><u>Extra locations on MRI:</u><br>None	**(PET/)CT:**<br>None<br><br>**MRI:**<br>None	**WGS:**<br>Not performed<br><br>**Histopathology:**<br>Adenocarcinoma (Lower GI tract)	**Diagnosis:**<br>Confirmed CUP<br><br>**Level of certainty:**<br>N/A (no diagnosis)<br><br>**Rationale:**<br>No tumour identified on imaging that would fit the histopathologic profile, negative colonoscopy.	☒ None<br>☐ Additional tumour sites detected<br>☐ Confirmed (PET/)CT diagnosis of primary tumour<br>☐ New diagnosis of primary tumour
8<br>71,M	Upper abdominal LN<br>Liver	<u>Known locations:</u><br>Fully concordant<br><br><u>Extra locations on MRI:</u><br>None	**(PET/)CT:**<br>None<br><br>**MRI:**<br>Liver (interpreted as primary tumour)	**WGS:**<br>86% Liver<br><br>WGS results available:<br>☒ Before MRI<br>☐ After MRI	**Diagnosis:**<br>Hepatocellular carcinoma(HCC)<br><br>**Level of certainty:**<br>☐ Possible (<50%)<br>☐ Probable (50-75%)<br>☒ Highly likely (>75%)<br><br>**Rationale:**<br>WGS results in concordance with imaging.	☐ None<br>☐ Additional tumour sites detected<br>☐ Confirmed (PET/)CT diagnosis of primary tumour<br>☒ New diagnosis of primary tumour<br>☒ In line with final diagnosis<br>☐ Not in line with final diagnosis

9<br>70,F	Lung<br>Anorectum	Known locations:<br>Concordant except for anorectum (benign on MRI)<br><br>Extra locations on MRI:<br>Galbladder<br><br>**(PET/)CT:**<br>None<br><br>**MRI:**<br>Gallbladder	**WGS:**<br>Not performed<br><br>**Histopathology:**<br>Mucinous adenocarcinoma (Lung, Proximal GI tract)	**Diagnosis:**<br>Confirmed CUP<br><br>**Level of certainty:**<br>N/A (no diagnosis)<br><br>**Rationale:**<br>Imaging findings not in line with histopathology; immune profile not typical for gallbladder or bile duct carcinoma.	☐ None<br>☒ Additional tumour sites detected<br>☐ Confirmed (PET/)CT diagnosis of primary tumour<br>☒ New diagnosis of primary tumour<br>☐ In line with final diagnosis<br>☒ Not in line with final diagnosis
10<br>71,M	lung<br>kidney (perirenal mass)	Known locations:<br>Concordant, but perirenal mass interpreted as possible duodenal mass<br><br>Extra locations on MRI:<br>None<br><br>**(PET/)CT:**<br>None<br><br>**MRI:**<br>Duodenum	**WGS:**<br>99% GI tract (small/large bowel or appendix)<br><br>WGS results available:<br>☐ Before MRI<br>☒ After MRI	**Diagnosis:**<br>Confirmed CUP<br><br>**Level of certainty:**<br>N/A (no diagnosis)<br><br>**Rationale:**<br>Following resection, it remained unclear whether the resected mass concerned an intestinal type urothelial carcinoma or a duodenal carcinoma.	☐ None<br>☐ Additional tumour sites detected<br>☐ Confirmed (PET/)CT diagnosis of primary tumour<br>☒ New diagnosis of primary tumour<br>☐ In line with final diagnosis<br>☒ Not in line with final diagnosis
11<br>64,F	Upper abdominal LN	Known locations:<br>Fully concordant<br><br>Extra locations on MRI:<br>Undefined duodenal or renal mass<br><br>**(PET/)CT:**<br>None<br><br>**MRI:**<br>Duodenum (or kidney / UCC)	**WGS:**<br>Not performed<br><br>**Histopathology:**<br>Signet ring cell adenocarcinoma (duodenum)	**Diagnosis:**<br>Small intestine cancer<br><br>**Level of certainty:**<br>☒ Possible (<50%)<br>☐ Probable (50-75%)<br>☐ Highly likely (>75%)	☐ None<br>☒ Additional tumour sites detected<br>☐ Confirmed (PET/)CT diagnosis of primary tumour

		Bone			**Rationale:**<br>Histopathology results in concordance with imaging findings.	☒ New diagnosis of primary tumour<br>☒ In line with final diagnosis<br>☐ Not in line with final diagnosis
12<br>74,F	Lung<br>Brain<br>Thyroid	<u>Known locations:</u><br>Concordant (Brain and thyroid not within MRI FOV)<br><br><u>Extra locations on MRI:</u><br>None	**(PET/)CT:**<br>None (possibly lung)<br><br>**MRI:**<br>None (possibly lung)	**WGS:**<br>Not performed<br><br>**Histopathology:**<br>Adenocarcinoma (Lung, GI tract, breast)	**Diagnosis:**<br>Confirmed CUP<br><br>**Level of certainty:**<br>N/A (no diagnosis)<br><br>**Rationale:**<br>No tumour identified on imaging that would fit the histopathologic profile.	☒ None<br>☐ Additional tumour sites detected<br>☐ Confirmed (PET/)CT diagnosis of primary tumour<br>☐ New diagnosis of primary tumour
13<br>70,M	Cervical LN<br>Upper abdominal LN<br>Lung<br>Duodenum	<u>Known locations:</u><br>Fully concordant<br><br><u>Extra locations on MRI:</u><br>None	**(PET/)CT:**<br>Duodenum<br><br>**MRI:**<br>Duodenum	**WGS:**<br>76% GI tract (small/large bowel or appendix)<br>12% Urothelial tract<br><br>WGS results available:<br>☐ Before MRI<br>☒ After MRI	**Diagnosis:**<br>Duodenal carcinoma<br><br>**Level of certainty:**<br>☐ Possible (<50%)<br>☐ Probable (50-75%)<br>☒ Highly likely (>75%)<br><br>**Rationale:**<br>WGS results in concordance with imaging findings.	☐ None<br>☐ Additional tumour sites detected<br>☒ Confirmed (PET/)CT diagnosis of primary tumour<br>☒ In line with final diagnosis<br>☐ Not in line with final diagnosis<br>☐ New diagnosis of primary tumour
14<br>79,F	Cervical LN<br>Thoracic LN<br>Upper abdominal LN<br>Peritoneum<br>Thoracic wall<br>Pleura (incl. effusion)	<u>Known locations:</u><br>Fully concordant<br><br><u>Extra locations on MRI:</u><br>None	**(PET/)CT:**<br>None<br><br>**MRI:**<br>None	**WGS:**<br>89% Ovary/Fallopian tube<br><br>WGS results available:<br>☐ Before MRI<br>☒ After MRI	**Diagnosis:**<br>Ovarian carcinoma<br><br>**Level of certainty:**<br>☐ Possible (<50%)<br>☐ Probable (50-75%)	☒ None<br>☐ Additional tumour sites detected<br>☐ Confirmed (PET/)CT diagnosis of primary tumour

				☒ Highly likely (>75%)<br><br>**Rationale:**<br>Histopathology revealed serous adenocarcinoma. Combined with the metastatic pattern and WGS, a primary (extra)ovarian carcinoma was suggested.	☐ New diagnosis of primary tumour
15<br>38,F	Cervical LN<br>Thoracic LN<br>Upper abdominal LN<br>Lung<br>Adrenal gland	<u>Known locations:</u><br>Concordant, except for upper abdominal LN (benign on MRI)<br><br><u>Extra locations on MRI:</u><br>None<br><br>**(PET/)CT:**<br>None (Possibly lung)<br><br>**MRI:**<br>None (Possibly lung)	**WGS:**<br>98% Lung: non-small cell<br><br>WGS results available:<br>☐ Before MRI<br>☒ After MRI	**Diagnosis:**<br>Lung carcinoma (NSCLC)<br><br>**Level of certainty:**<br>☐ Possible (<50%)<br>☐ Probable (50-75%)<br>☒ Highly likely (>75%)<br><br>**Rationale:**<br>WGS results in concordance with (possible) imaging findings.	☒ None<br>☐ Additional tumour sites detected<br>☐ Confirmed (PET/)CT diagnosis of primary tumour<br>☐ New diagnosis of primary tumour
16<br>73,F	Thoracic LN<br>Liver<br>Bone<br>Pleural effusion	<u>Known locations:</u><br>Fully concordant<br><br><u>Extra locations on MRI:</u><br>Peritoneum<br>Gallbladder<br><br>**(PET/)CT:**<br>None<br><br>**MRI:**<br>Gallbladder (with perforation)	**WGS:**<br>Not performed<br><br>**Histopathology before MRI:**<br>Adenocarcinoma (Pancreas/bile ducts, proximal GI tract, lung)	**Diagnosis:**<br>Pancreatic cancer<br><br>**Level of certainty:**<br>☐ Possible (<50%)<br>☐ Probable (50-75%)<br>☒ Highly likely (>75%)<br><br>**Rationale:**<br>Gastroscopy with EUS revealed a pancreatic lesion, which was biopsied (PA: adenocarcinoma).	☐ None<br>☒ Additional tumour sites detected<br>☐ Confirmed (PET/)CT diagnosis of primary tumour<br>☒ New diagnosis of primary tumour<br>☐ In line with final diagnosis<br>☒ Not in line with final diagnosis

				Gallbladder tuned out to be perforated cholecystitis.	
17<br>73,M	Cervical LN<br>Thoracic LN<br>Upper abdominal LN<br>Liver<br>Lung	<u>Known locations:</u><br>Fully concordant<br><br><u>Extra locations on MRI:</u><br>Pancreas (misinterpreted as lymph node on PET)<br><br>**(PET/)CT:**<br>None<br><br>**MRI:**<br>Pancreas	**WGS:**<br>26% Kidney<br>22% Pancreas<br>10% Bone/Soft tissue<br><br>WGS results available:<br>☐ Before MRI<br>☒ After MRI	**Diagnosis:**<br>Pancreatic cancer<br><br>**Level of certainty:**<br>☐ Possible (<50%)<br>☐ Probable (50-75%)<br>☒ Highly likely (>75%)<br><br>**Rationale:**<br>A pancreatic mass was seen on MRI (in retrospect also visible on PET). WGS confirmed possibility of a primary pancreatic cancer.	☐ None<br>☒ Additional tumour sites detected<br>☐ Confirmed (PET/)CT diagnosis of primary tumour<br>☒ New diagnosis of primary tumour<br>☒ In line with final diagnosis<br>☐ Not in line with final diagnosis
18<br>62,F	Thoracic LN<br>Upper abdominal LN<br>Lower abdominal LN<br>Inguinal LN<br>Pleura	<u>Known locations:</u><br>Fully concordant<br><br><u>Extra locations on MRI:</u><br>Ovary<br><br>**(PET/)CT:**<br>None<br><br>**MRI:**<br>Ovary	**WGS:**<br>65% Ovary/Fallopian tube<br>33% Breast<br><br>WGS results available:<br>☒ Before MRI<br>☐ After MRI	**Diagnosis:**<br>Ovarian carcinoma<br><br>**Level of certainty:**<br>☐ Possible (<50%)<br>☐ Probable (50-75%)<br>☒ Highly likely (>75%)<br><br>**Rationale:**<br>WGS results in concordance with imaging findings.	☐ None<br>☒ Additional tumour sites detected<br>☐ Confirmed (PET/)CT diagnosis of primary tumour<br>☒ New diagnosis of primary tumour<br>☒ In line with final diagnosis<br>☐ Not in line with final diagnosis

19<br>61,M	Thoracic LN<br>Upper abdominal LN<br>Liver<br>Adrenal gland<br>Lung<br>Kidney<br>Peritoneum	Known locations:<br>Fully concordant<br><br>Extra locations on MRI:<br>None	**(PET/)CT:**<br>Kidney or lung<br><br>**MRI:**<br>Kidney	**WGS:**<br>96% GI tract (small/large bowel or appendix)<br><br>WGS results available:<br>☒ Before MRI<br>☐ After MRI	**Diagnosis:**<br>Confirmed CUP<br><br>**Level of certainty:**<br>N/A (no diagnosis)<br><br>**Rationale:**<br>Imaging findings (possible kidney or lung tumour) not in line with WGS. Negative colonoscopy.	☐ None<br>☐ Additional tumour sites detected<br>☒ Confirmed (PET/)CT diagnosis of primary tumour<br>☐ In line with final diagnosis<br>☒ Not in line with final diagnosis<br>☐ New diagnosis of primary tumour
20<br>81,F	Cervical LN<br>Upper abdominal LN	Known locations:<br>Fully concordant<br><br>Extra locations on MRI:<br>Kidney	**(PET/)CT:**<br>None<br><br>**MRI:**<br>None (possibly kidney)	**WGS:**<br>53% GI tract (small/large bowel or appendix)<br>37% Endometrium<br><br>WGS results available:<br>☒ Before MRI<br>☐ After MRI	**Diagnosis:**<br>Confirmed CUP<br><br>**Level of certainty:**<br>N/A (no diagnosis)<br><br>**Rationale:**<br>Imaging findings (possible kidney tumour) not in line with WGS. Negative colonoscopy.	☐ None<br>☒ Additional tumour sites detected<br>☐ Confirmed (PET/)CT diagnosis of primary tumour<br>☐ New diagnosis of primary tumour
21<br>82,M	Bone	Known locations:<br>Fully concordant<br><br>Extra locations on MRI:<br>None	**(PET/)CT:**<br>None<br><br>**MRI:**<br>None	**WGS:**<br>54% Bile duct/gallbladder<br>12% Kidney<br>11% Mesothelium<br><br>WGS results available:<br>☒ Before MRI<br>☐ After MRI	**Diagnosis:**<br>Confirmed CUP<br><br>**Level of certainty:**<br>N/A (no diagnosis)<br><br>**Rationale:**<br>No evidence of primary biliary tumour, renal tumour or mesothelioma on imaging, as suggested by WGS .	☒ None<br>☐ Additional tumour sites detected<br>☐ Confirmed (PET/)CT diagnosis of primary tumour<br>☐ New diagnosis of primary tumour

22<br>78,M	Upper abdominal LN<br>Liver<br>Duodenum<br>Peritoneum	<u>Known locations:</u> Fully concordant<br><br><u>Extra locations on MRI:</u> None<br><br>**(PET/)CT:** None<br><br>**MRI:** Duodenum	**WGS:**<br>98% Pancreas<br><br>WGS results available:<br>☐ Before MRI<br>☒ After MRI	**Diagnosis:**<br>Pancreatic cancer<br><br>**Level of certainty:**<br>☐ Possible (<50%)<br>☒ Probable (50-75%)<br>☐ Highly likely (>75%)<br><br>**Rationale:**<br>Suspected duodenal mass located adjacent to the pancreatic head. Following WGS, images were re-evaluated and mass was re-interpreted as potentially originating from the pancreas.	☐ None<br>☐ Additional tumour sites detected<br>☐ Confirmed (PET/)CT diagnosis of primary tumour<br>☒ New diagnosis of primary tumour<br>☐ In line with final diagnosis<br>☒ Not in line with final diagnosis
23<br>64,M	Brain<br>Cervical LN<br>Upper abdominal LN<br>Lower abdominal LN<br>Ascites<br>Muscle (leg)	<u>Known locations:</u> Fully concordant (muscle lesion not within MRI FOV)<br><br><u>Extra locations on MRI:</u> None<br><br>**(PET/)CT:** None<br><br>**MRI:** None	**WGS:**<br>52% GI tract (small/large bowel or appendix)<br>24% Urothelial tract<br><br>WGS results available:<br>☒ Before MRI<br>☐ After MRI	**Diagnosis:**<br>Sigmoid carcinoma<br><br>**Level of certainty:**<br>☐ Possible (<50%)<br>☐ Probable (50-75%)<br>☒ Highly likely (>75%)<br><br>**Rationale:**<br>Colonoscopy revealed a sigmoid tumour, which was confirmed by biopsy.	☒ None<br>☐ Additional tumour sites detected<br>☐ Confirmed (PET/)CT diagnosis of primary tumour<br>☐ New diagnosis of primary tumour
24<br>71,M	Cervical LN<br>Upper abdominal LN<br>Bladder/Urinary tract	<u>Known locations:</u> Lymph nodes concordant, urinary tract benign on MRI<br><br><u>Extra locations on MRI:</u> None<br><br>**(PET/)CT:** None<br><br>**MRI:** None	**WGS:**<br>33% Bile duct/Gallbladder<br>22% GI tract (small/large bowel or appendix)<br><br>WGS results available:	**Diagnosis:**<br>Confirmed CUP<br><br>**Level of certainty:**<br>N/A (no diagnosis)<br><br>**Rationale:**	☒ None<br>☐ Additional tumour sites detected<br>☐ Confirmed (PET/)CT diagnosis of primary tumour<br>☐ New diagnosis of primary tumour

				☐ Before MRI<br>☒ After MRI	No tumour identified on imaging that would fit the WGS results.	
25<br>67,F	Duodenum<br>Peritoneum<br>Upper abdominal LN	<u>Known locations:</u><br>Fully concordant<br><br><u>Extra locations on MRI:</u><br>None	**(PET/)CT:**<br>Possibly duodenum<br><br>**MRI:**<br>Duodenum	**WGS:**<br>61% GI tract (small/large bowel or appendix)<br>35% Upper GI tract<br><br>**WGS results available:**<br>☐ Before MRI<br>☒ After MRI	**Diagnosis:**<br>Small intestine (duodenal) carcinoma<br><br>**Level of certainty:**<br>☐ Possible (<50%)<br>☐ Probable (50-75%)<br>☒ Highly likely (>75%)<br><br>**Rationale:**<br>WGS results in concordance with imaging findings.	☐ None<br>☐ Additional tumour sites detected<br>☒ Confirmed (PET/)CT diagnosis of primary tumour<br>☒ In line with final diagnosis<br>☐ Not in line with final diagnosis<br>☐ New diagnosis of primary tumour
26<br>65,M	Peritoneum	<u>Known locations:</u><br>Fully concordant<br><br><u>Extra locations on MRI:</u><br>Appendix	**(PET/)CT:**<br>None<br><br>**MRI:**<br>Appendix	**WGS:**<br>Not performed<br><br>**Histopathology:**<br>Mucinous neoplasia (appendix, colon)	**Diagnosis:**<br>Appendiceal carcinoma<br><br>**Level of certainty:**<br>☐ Possible (<50%)<br>☐ Probable (50-75%)<br>☒ Highly likely (>75%)<br><br>**Rationale:**<br>MRI findings (appendiceal lesion) and histopathology (mucinous tumour) in combination with peritoneal disease.	☐ None<br>☒ Additional tumour sites detected<br>☐ Confirmed (PET/)CT diagnosis of primary tumour<br>☒ New diagnosis of primary tumour<br>☒ In line with final diagnosis<br>☐ Not in line with final diagnosis

27<br>33,M	Cervical LN<br>Thoracic LN<br>Upper abdominal LN<br>Lower abdominal LN<br>Lung<br>Penis	<u>Known locations:</u><br>Concordant except for penis lesion (benign on MRI)<br><br><u>Extra locations on MRI:</u><br>Intrahepatic bile ducts	**(PET/)CT:**<br>Penis<br><br>**MRI:**<br>Intrahepatic bile ducts	**WGS:**<br>43% Pancreas<br>22% Esophagus/Stomach<br><br>WGS results available:<br>☒ Before MRI<br>☐ After MRI	**Diagnosis:**<br>Confirmed CUP<br><br>**Level of certainty:**<br>N/A (no diagnosis)<br><br>**Rationale:**<br>Imaging findings (possible penile or bile duct tumour) not in line with WGS.	☐ None<br>☒ Additional tumour sites detected<br>☐ Confirmed (PET/)CT diagnosis of primary tumour<br>☒ New diagnosis of primary tumour<br>☐ In line with final diagnosis<br>☒ Not in line with final diagnosis
28<br>47,F	Liver<br>Upper abdominal LN	<u>Known locations:</u><br>Fully concordant<br><br><u>Extra locations on MRI:</u><br>None	**(PET/)CT:**<br>None<br><br>**MRI:**<br>None (one of the liver lesions could theoretically represent the primary tumour)	**WGS:**<br>17% melanoma<br>16% Bile duct/gallbladder<br>14% Breast<br><br>WGS results available:<br>☒ Before MRI<br>☐ After MRI	**Diagnosis:**<br>Intrahepatic cholangiocarcinoma<br><br>**Level of certainty:**<br>☒ Possible (<50%)<br>☐ Probable (50-75%)<br>☐ Highly likely (>75%)<br><br>**Rationale:**<br>WGS findings combined with possible intrahepatic bile duct mass on MRI.	☒ None<br>☐ Additional tumour sites detected<br>☐ Confirmed (PET/)CT diagnosis of primary tumour<br>☐ New diagnosis of primary tumour
29<br>67,F	Thoracic LN	<u>Known locations:</u><br>Fully concordant<br><br><u>Extra locations on MRI:</u><br>Breast	**(PET/)CT:**<br>None<br><br>**MRI:**<br>Breast	**WGS:**<br>97% Breast<br><br>WGS results available:<br>☒ Before MRI<br>☐ After MRI	**Diagnosis:**<br>Breast cancer<br><br>**Level of certainty:**<br>☐ Possible (<50%)<br>☐ Probable (50-75%)<br>☒ Highly likely (>75%)<br><br>**Rationale:**<br>Concordant findings on WGS and MRI.	☐ None<br>☐ Additional metastatic sites<br>detected<br>☐ Confirmed (PET/)CT diagnosis of primary tumour<br>☒ New diagnosis of primary tumour<br>☒ In line with final diagnosis

						☐ Not in line with final diagnosis
30<br>61,F	Upper abdominal LN<br>Lower abdominal NL<br>Liver<br>Peritoneum<br>Possibly ovary	<u>Known locations:</u><br>Concordant except for ovary (benign on MRI)<br><br><u>Extra locations on MRI:</u><br>None	**(PET/)CT:**<br>None<br><br>**MRI:**<br>None	**WGS:**<br>99% GI tract (small/large bowel or appendix)<br><br>WGS results available:<br>☐ Before MRI<br>☒ After MRI	**Diagnosis:**<br>Colorectal carcinoma<br><br>**Level of certainty:**<br>☐ Possible (<50%)<br>☐ Probable (50-75%)<br>☒ Highly likely (>75%)<br><br>**Rationale:**<br>WGS results combined with histologically proven colorectal adenocarcinoma (note: in polyp that had already been resected prior to imaging).	☒ None<br>☐ Additional metastatic sites detected<br>☐ Confirmed (PET/)CT diagnosis of primary tumour<br>☐ New diagnosis of primary tumour
